# Laparoscopic treatment of an unruptured cystic artery pseudo-aneurysm in the presence of calculous emphysematous cholecystitis

**DOI:** 10.1093/jscr/rjab625

**Published:** 2022-01-31

**Authors:** Sarah Kelly, Krisha Shah, Jasneet Motizada, Fiametta Soggiu, Hemant Sheth

**Affiliations:** Department of General Surgery, Ealing Hospital, London Northwest NHS Healthcare Trust, London, UK

## Abstract

Cystic artery pseudoaneurysm is a rare complication of invasive biliary procedures or of acute or chronic cholecystitis and pancreatitis. Emphysematous cholecystitis is an acute inflammatory process of the gallbladder due to gas forming organisms such as *Escherichia coli* and *Clostridium perfringens*. We report the case of a 34-year-old gentleman admitted with a 3-day history of generalized abdominal pain, vomiting and markedly raised inflammatory markers. A computed tomography scan demonstrated acute calculus cholecystitis and an incidental CAP. This was successfully treated with an emergency laparoscopic cholecystectomy. CAPs are reported in the literature as rare and are usually diagnosed after rupture with severe haemorrhage. In this report, we highlight that a non-ruptured CAP identified preoperatively can be safely managed simultaneously with a laparoscopic approach, thus avoiding the need for invasive angiographic procedures or open surgery.

## INTRODUCTION

A pseudoaneurysm is an abnormal dilatation of an artery secondary to trauma or an inflammatory process. Cystic artery pseudoaneurysm (CAP) is a rare entity with a limited number of cases noted worldwide, with ~25 cases reported by Loizides *et al*. [1] . It is primarily known to be a complication of biliary surgery, and furthermore, has been seen after radiological biliary procedures such as endoscopic retrograde cholangiopancreatography [2]. In rare cases, it has also been shown to be a consequence of acute or chronic cholecystitis or pancreatitis [3]. CAP has occasionally been seen as a sequelae of local vascular inflammation from acute or chronic cholecystitis; however, to our knowledge, there has not been any documentation of emphysematous cholecystitis (EC) causing CAP [4].

Patients with CAPs most frequently present with rupture and therefore often experience symptoms such as haemobilia, haematemesis, melena and severe abdominal pain [5]. Haemobilia itself is noted to have its own triad of symptoms, known as Quincke’s triad, including jaundice, right upper quadrant pain and upper gastrointestinal haemorrhage. Previous ruptured CAPs have successfully been treated with cholecystectomy and ligation of the cystic artery [6].

We present the case of a young patient presenting with EC and an incidental finding on preoperative imaging an unruptured cystic artery pseudoaneurysm.

## CASE REPORT

This 34-year-old European male was referred from A&E after complaining of a 3-day history of generalized abdominal pain, nausea, vomiting and constipation. He was unable to eat and drink due to the intensity of the pain. He was otherwise a fit and well patient with no previous medical history and an open appendicectomy at age 9. He was an active smoker, with occasional alcohol intake and a normal body mass index.

On initial examination, the patient was obviously in discomfort with no clinical signs of jaundice. On palpation of the abdomen, he was tender in the epigastrium, with guarding in the right flank and right iliac fossa but no rebound tenderness. No organomegaly was noted and bowel sounds were present. A Lanz appendicectomy scar was noted.

**Figure 1:**
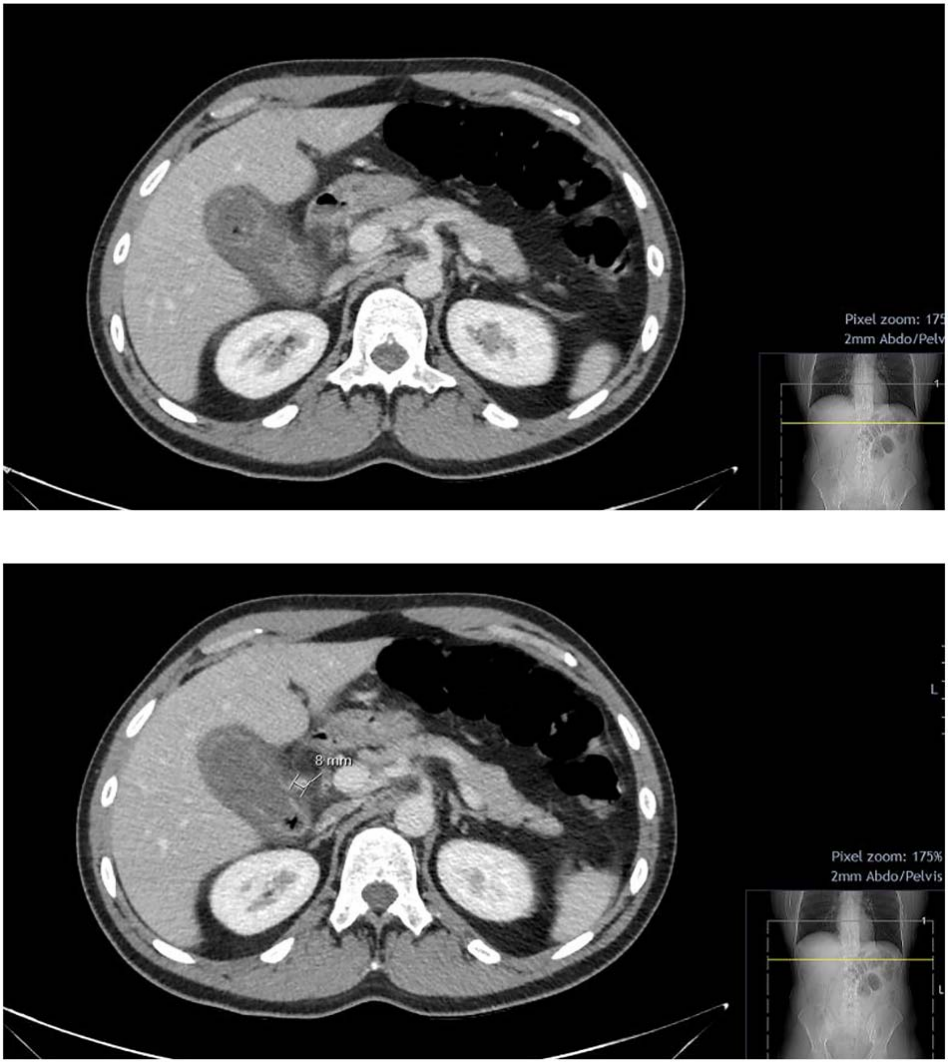
CT abdomen and pelvis with contrast; hyper-enhancing, oedematous gallbladder wall with fat stranding and gas locules consistent with acute calculous cholecystitis; suspected pseudoaneurysm of the cystic artery measuring 7.6 mm.

On initial blood tests, he had a raised inflammatory markers (C-reactive protein: 201 mg/l, white cell count: 16.2x10^9^/l), a slightly raised ALP (99 IU/l) however all other blood results were unremarkable. He underwent a computed tomography (CT) abdomen pelvis with contrast, which showed a picture of acute calculous cholecystitis with oedematous walls and air within the walls and the gallbladder itself, raising a suspicion of EC. Incidentally, a 7.6-mm pseudoaneurysm was noted at the level of the anterior branch of the cystic artery (Figs 1 and 2).

The patient was haemodynamically stable despite the ongoing sepsis, and there was no clinical suspicion of haemorrhage; therefore, we proceeded with an emergency laparoscopic cholecystectomy. The intraoperative findings confirmed gangrenous cholecystitis. A careful dissection at the Calot’s triangle allowed for identification of the CAP. The procedure was performed with the standard four ports technique. The dissection allowed to achieve a critical view of safety and the CAP was pre-emptively excluded with two proximal Hem-o-lok green clips. The intraoperative estimated blood loss was around 300 ml, relating to the severe inflammatory process. A percutaneous through right lateral port was left *in situ*.

The patient was monitored in the high-dependency unit for the first 24 hours. His post-operative course was uneventful and was discharged home after completing a course of antibiotics and removal of the drain.

The histology report showed extensive acute necrotic cholecystitis with widespread ulceration, haemorrhage, abscesses and eosinophils. Bile cultures were sent and showed no significant bacterial growth.

## DISCUSSION

In the majority of cases, CAP is noted to be a complication of biliary surgery. In this case, however, the CAP is thought to be a consequence of acute calculous cholecystitis. A patient with a ruptured CAP usually presents with clinical signs of shock, rarely haemobilia and the Quincke’s triad.

Unruptured CAPs are extremely rare, with only two cases previously reported [1, 7] and may be incidentally diagnosed on a preoperative CT scan. Although the exact mechanism of a CAP is not known, it has been postulated that the cystic artery is eroded by the direct pressure of the gallstones or by the inflammation of the arterial wall. Subsequently, there is damage to the adventitia leading to vessel wall weakness, resulting in a pseudoaneurysm [1, 7].

In this case, the patient presented with an EC which could have led to the CAP. Literature states that this disease is more common in older patients and in presence of concomitant pathologies such as poorly controlled diabetes mellitus, immune suppression and peripheral vascular disease. The patient in this case had no such risk factors identified [8].

The most sensitive way to diagnose EC is by CT due to the presence of intraluminal or intramural gas in the gall bladder and bile ducts. The CT scan can also reveal pre-cholecystic inflammatory changes. Early diagnosis and treatment are essential, since if left untreated, EC can progress to soft tissue gangrene and lead to sepsis and death [9]. In this case, the additional finding of a CAP requested a prompt treatment strategy to avoid the risk of further complications in the case of acute rupture.

Being a rare entity, there are no official guidelines on the management of CAP. Historically, CAP was managed by open cholecystectomy with ligation of the aneurysm. Some authors reported cases of ruptured CAP treated with coeliac arteriography, selective embolization and coiling [10]. Our case, along with a few other case reports, successfully demonstrated that a laparoscopic approach is a suitable intervention for the management of unruptured CAP [11].

## CONCLUSION

In conclusion, CAPs are rare, with unruptured CAPs being even more of a rare entity. To our knowledge, excluding our case, there were only four cases with unruptured aneurysms [12]. Historically, CAP was managed by open cholecystectomy; however, this case successfully demonstrates that a laparoscopic approach is a suitable intervention of unruptured CAP.

## CONFLICT OF INTEREST STATEMENT

None declared.

## FUNDING

None.
